# The Mitochondrion as Potential Interface in Early-Life Stress Brain Programming

**DOI:** 10.3389/fnbeh.2018.00306

**Published:** 2018-12-06

**Authors:** Anke Hoffmann, Dietmar Spengler

**Affiliations:** Epigenomics of Early Life, Translational Research in Psychiatry, Max Planck Institute of Psychiatry, Munich, Germany

**Keywords:** early-life stress, brain programming, mitochondria, bioenergetics, steroidogenesis, oxidative stress, placenta, peripheral blood cells

## Abstract

Mitochondria play a central role in cellular energy-generating processes and are master regulators of cell life. They provide the energy necessary to reinstate and sustain homeostasis in response to stress, and to launch energy intensive adaptation programs to ensure an organism’s survival and future well-being. By this means, mitochondria are particularly apt to mediate brain programming by early-life stress (ELS) and to serve at the same time as subcellular substrate in the programming process. With a focus on mitochondria’s integrated role in metabolism, steroidogenesis and oxidative stress, we review current findings on altered mitochondrial function in the brain, the placenta and peripheral blood cells following ELS-dependent programming in rodents and recent insights from humans exposed to early life adversity (ELA). Concluding, we propose a role of the mitochondrion as subcellular intersection point connecting ELS, brain programming and mental well-being, and a role as a potential site for therapeutic interventions in individuals exposed to severe ELS.

## Introduction

Early life experiences can cause lasting changes in brain structure and function. This process is often referred to as developmental programming (Gluckman and Hanson, 2004) and evolves from developmental plasticity. Accordingly, a single genotype gives rise to different morphological and/or physiological phenotypes in response to experiences made during sensitive time windows.

Social experiences are among the most powerful to elicit plastic changes in the human brain and to shape structure and function of circuitries underlying social and emotional behavior (Davidson and McEwen, 2012). Early experiences in this domain are also likely to govern differences among individuals in their vulnerability or resilience to future adversity (Hochberg et al., 2011). For example, parental maltreatment (e.g., physical or emotional abuse and/or neglect) associates with early-life stress (ELS) in the infant (Murgatroyd and Spengler, 2011b; Noll and Shalev, 2018) that profoundly influences the experience-dependent maturation of structures underlying emotional and endocrine responses to stress. ELS programs hippocampal and hypothalamo-pituitary-adrenal (HPA) axis functions—integral components of the body’s stress response—and leads to increased stress responsivity in adulthood (Murgatroyd and Spengler, 2011a; Nemeroff, 2016). Likewise, maternal exposure to adversity during pregnancy impacts the quality of fetal growth and development and enhances the risk for disturbed HPA-axis regulation in the offspring, a known risk factor for various psychiatric disorders such as schizophrenia or major depression (O’Donnell and Meaney, 2017).

ELS-dependent programming of the brain takes place at different functional layers. Studies in animals have highlighted structural changes consisting of alterations in spine density, dendritic length and branching in relevant brain regions such as the hippocampus, prefrontal cortex (PFC) and amygdala (Lupien et al., 2009). Relatedly, ELS-induced programming can lead to changes in cell numbers such as diminished neurogenesis in the adult dentate gyrus (Karten et al., 2005). A recent leap forward in our understanding of the molecular basis of programming relates to the advent of epigenetics. This research field has identified mechanisms such as DNA-methylation, chromatin modifications and non-coding RNAs, among others that establish long-lasting changes in transcriptional programs governing social, emotional and neuroendocrine functions in response to ELS and thus serve as a bridge between experience and behavioral change (Murgatroyd et al., 2010; Zhang and Meaney, 2010; Hochberg et al., 2011; Hoffmann and Spengler, 2014; Cao-Lei et al., 2017).

Notwithstanding this progress, there is little doubt that additional layers of programming are likely to exist given the highly complex organization of mammalian cells. As the smallest unit of life, cells harbor different organelles that are specialized for carrying out one or more vital functions, analogous to the organs of the human body (such as the heart, lung, kidney and so forth with each organ performing a different function). Organelles such as the nucleus and golgi apparatus are typically solitary, while others such as the mitochondria, peroxisomes and lysosomes are present in numbers of hundreds to thousands (Alberts et al., 2014). While nuclear responses (i.e., changes in DNA methylation and chromatin modification) have caught mounting attention over the last years, each of these organelles and their tightly interwoven interactions are vital to cellular function and could serve as subcellular substrate in developmental programming. To explore this hypothesis, we will discuss in this review article the role of the mitochondrion as a multifunctional life-sustaining organelle and potential intersection point in ELS-induced brain programming. The principle theme that will be presently developed is how mitochondria respond and adapt to stress, and whether mitochondrial function couples to ELS-induced brain programming. At the same time, we consider the possibility that mitochondria are not only the mediator, but also the target of ELS-dependent brain programming.

We explain first origin and structure of the mitochondrion to proceed from there to the question how mitochondrial bioenergetics, steroidogenesis and reactive oxygen species (ROS) production could underpin ELS-induced programming. Following this, we examine current evidence from animal and human studies for a role of the mitochondrion to couple to and serve in ELS-dependent programming of the brain.

## Methods

The literature selection process for this review was performed in the databank PubMed and included combinations of the following search terms: stress* (e.g., stress, stressful and stressor), early life* (e.g., stress, adversity and maltreatment) and “mitochondri*” (e.g., mitochondrion, mitochondria and mitochondrial). Date limits were from 1970 to June 2018. Only studies in English that measured at least one aspect of mitochondrial *function* (e.g., bioenergetics, steroidogenesis, respiratory chain activity and ROS production), or *of* mitochondrial morphology and structure were included. Following this, additional searches included scrutiny of references from the identified publications, of similar articles suggested by PubMed and of citatory publications using Google Scholar^®^.

## Mitochondrion’s Past and Today’s Body Plan

**Figure 1:**
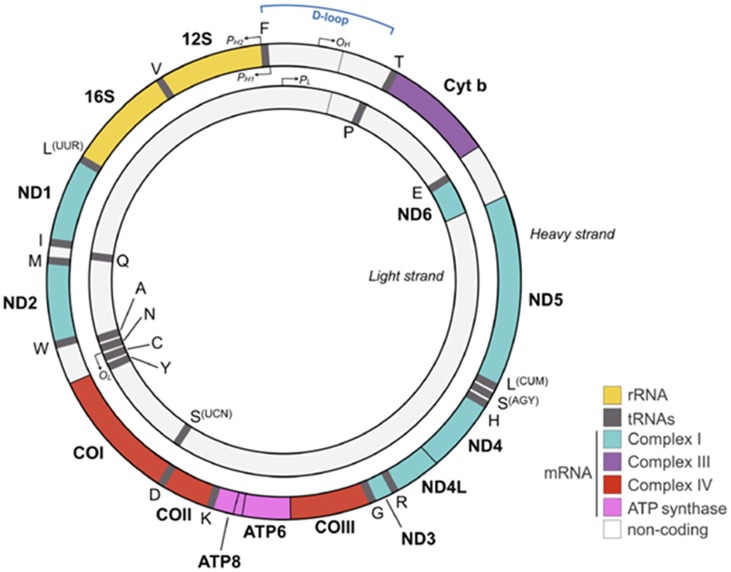
Human mitochondrial DNA (mtDNA). The mtDNA consists of a light (inner) and heavy (outer) circular DNA strand with each containing a separate origin of replication termed O_L_ and O_H_, respectively. The mtDNA comprises 16,569 nucleotides and encodes 37 genes, including 2 ribosomal and 22 transport RNAs required for protein synthesis. Mitochondrial encoded proteins constitute essential parts of the respiratory chain by contributing seven subunits to complex I, one subunit to complex III, three subunits to complex IV, and two subunits to complex V (adenosine triphosphate, ATP synthase). Promoters on the heavy strand (P_H1_ and P_H2_) and on the light strand (P_L_) drive mtDNA gene expression. The glucocorticoid receptor (GR) binds to the mtDNA near the D-loop straddling the O_H_ origin. Colors for each gene match the respiratory chain complexes shown in Figures 2, 5. 1 is adapted from Picard and McEwen (2018) license number 4360110364852.

Approximately 2 billion years ago, one of our single-celled ancestors engulfed an oxygen-consuming bacterium that became the mitochondrion capable of producing large amounts of energy in the form of adenosine triphosphate (ATP). Each cell harbors hundreds to thousands of these organelles with each possessing its own mitochondrial DNA (mtDNA), RNA and protein synthesis system. Reflecting its bacterial origin, the mitochondrion holds 5–10 mtDNA copies in form of a double stranded, closed circular and maternally inherited DNA (Figure 1).

The significantly larger sized mtDNA in our proto-eukaryotic ancestor has been reduced by the transfer of mtDNA encoded genes to the cellular nucleus. As a result of this transfer, today’s mtDNA encodes only 13 polypeptides, two rRNAs (12S and 16S) and 22 tRNAs that are essential for the oxidative phosphorylation system (OXPHOS; Figure 1). By contrast, the cell’s nuclear genome encodes some 1,500 proteins contributing to OXPHOS.

**Figure 2:**
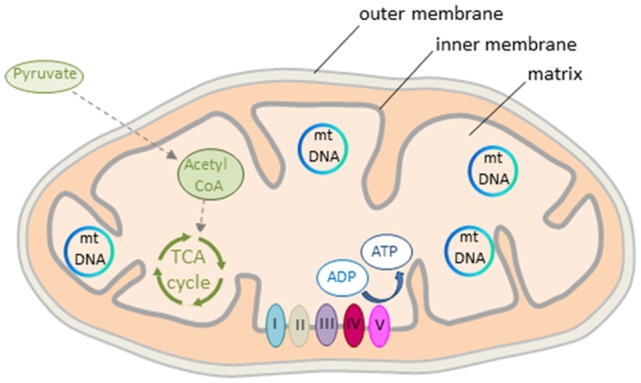
Mitochondrion’s structure and energy production. The mitochondrion consists of outer and inner double-layered membranes enclosing the intermembrane space, the cristae space formed by infoldings of the inner membrane, and the matrix as the space within the inner membrane. The matrix hosts the circular mtDNA and a large number of enzymes catalyzing various biochemical reactions. Acetyl CoA, a reaction product of pyruvate, is the major substrate fueling the tricarboxylic acid (TCA) cycle in early life. Oxidation of pyruvate produces reduced cofactors that transfer free electrons to the respiratory chain situated in the inner mitochondrial membrane. The respiratory chain consists of complex I to V that build the mitochondrial membrane potential. This is then used for ATP synthesis. Colors from respiratory complexes match the contribution of mitochondrial gene products shown in Figure 1.

The mitochondrion is a double membrane-bound organelle present in all mammalian cells except erythrocytes (Figure 2). The matrix contains a concentrated mixture of enzymes catalyzing the oxidation of acetyl CoA (see further below) via the tricarboxylic acid (TCA) cycle. Electrons derived from reduced cofactors are passed through the respiratory chain (also referred to as electron transport chain, ETC) situated in the inner membrane (Figure 2). This process generates an electrical charge across the inner membrane termed mitochondrial membrane potential (Δψm). The membrane potential is then used to produce energy in the form of ATP via the ATP synthase to empower various cellular functions including neuronal activity (see further below).

Mitochondria are dynamic organelles with the ability to undergo fusion or fission and form constantly changing tubular networks in response to metabolic demand or environmental stress. An increase in fusion activity leads to mitochondrial elongation (increasing mitochondrial capacity), whereas an increase in fission activity results in mitochondrial fragmentation and the release of pro-apoptotic components (decreasing mitochondrial capacity).

## Mitochondria at the Interface of Stress and Adaptation

The human brain consumes 20% of the body’s oxygen uptake even though it accounts for only 2% of its weight (Rolfe and Brown, 1997). Neurons account for most (≈80%–90%) of the energy demand of the brain (Yu et al., 2017); for example, cortical neurons consume in the resting state approximately 4.7 billion molecules of ATP per second with the highest energy demand around the synapse (Zhu et al., 2012). Stress, including complex social interactions, strongly enhances the brain’s energy demand (Bryan, 1990) by setting off short- and long-term changes in cellular composition, structure and function that underpin behavioral and physiological adaptations that must be matched to the available supply of energy (Picard et al., 2014). The secretion of glucocorticoids (GCs) and catecholamines leads to the mobilization of large amounts of substrates from the body’s energy stores that are metabolized in the mitochondria via the TCA cycle (also referred to as citric acid cycle or Krebs cycle) that contributes to over 90% of the cellular energy production.

**Figure 3:**
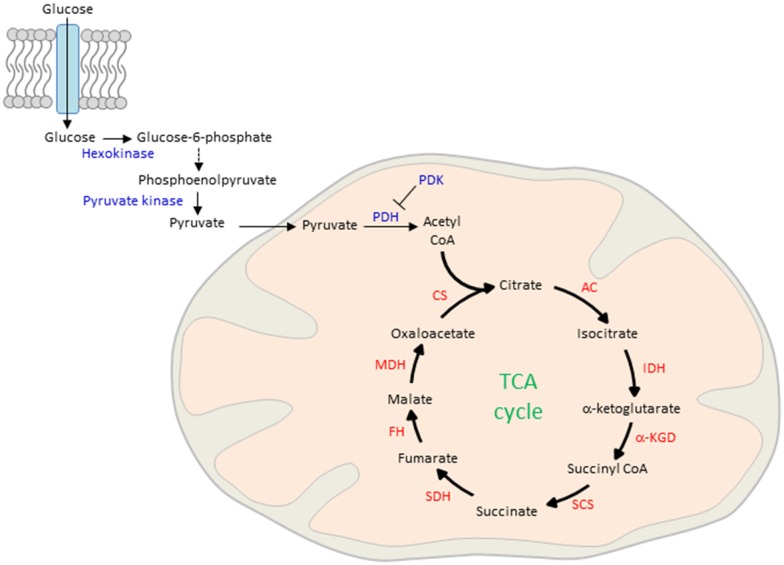
Glucose uptake and mitochondrial oxidation. Blood-borne glucose is taken up by cells through membranous glucose transporters. In the cytoplasm, glucose is phosphorylated by hexokinase and further metabolized by a series of enzymatic reactions to phosphoenolpyruvate. This reaction product is phosphorylated by pyruvate kinase and actively transported into the mitochondrial matrix. There, pyruvate is oxidized by pyruvate dehydrogenase (PDH) to form acetyl CoA, the major fuel of the TCA in early life. On the other hand, pyruvate dehydrogenase kinase (PDK) inhibits pyruvate oxidation in peripheral tissues. CS, citrate synthase; AC, aconitase; IDH, isocitrate dehydrogenase; α-KGD, α-ketoglutarate-dehydrogenase; SCS, succinyl-CoA synthetase; SDH, succinite dehydrogenase; FH, fumarase; MDH, malate dehydrogenase.

The process of ATP production begins with the lysis of glucose in the cytosol and the active transport of the reaction product pyruvate into the mitochondrial matrix (Figure 3). There, pyruvate is oxidized by pyruvate dehydrogenase (PDH) and combines with coenzyme A to form acetyl CoA. Although glucose is the main obligatory energy substrate for the adult brain and is also very important for the developing brain (McKenna et al., 2012), other substrates, mainly ketone bodies and lactate can be used in addition during normal development for energy production and biosynthesis of proteins and lipids. As a result of the high fat content of maternal milk (Schousboe and Sonnewald, 2016), the rate of uptake and metabolism of the two ketone bodies β-hydroxybutyrate and acetoacetate is high during lactation. In the mitochondrion, β-hydroxybutyrate is successively degraded in the end product acetyl-CoA, the first intermediate in common with the pathway of glucose metabolism. Acetoacetate can be metabolized either in the mitochondrion by the same pathway as β-hydroxybutyrate or can be also metabolized in the cytosol. Ketone body metabolism in the immature brain supports cell growth, building of cell membranes and organelles, and myelination (Schousboe and Sonnewald, 2016). Additionally, it serves to spare glucose for metabolic pathways that cannot be fueled by ketones such as the pentose phosphate pathway, which leads to the biosynthesis of ribose mandatory for DNA synthesis and of nicotinamide adenosine dinucleotide phosphate (NADPH), a key cofactor in lipid biosynthesis. Spared glucose is necessary to support emerging sensory functions as well as more integrated and adapted behaviors whose establishment during brain maturation seems to critically depend on the active supply and site-specific increased use of glucose. Beyond ketone body metabolism, anaplerotic reactions do replenish TCA cycle intermediates that have been extracted for biosynthesis and contribute thus to acetyl CoA formation (Schousboe and Sonnewald, 2016). For example, the anaplerotic pathway from propionyl-CoA via methylmalonyl-CoA to succinyl-CoA operates in both peripheral tissues and the brain. Anaplerotic molecules metabolized to propionyl-CoA and the propionyl-CoA carboxylase pathway include the branched chain amino acids, isoleucine and valine, propionate and molecules containing fatty acids with an uneven number of carbon atoms, such as triheptanoin. However, anaplerotic reactions match cell-type and tissue specific demands and can also differ between peripheral and central tissues. While glutamine, the most abundant circulating amino acid, serves in peripheral tissues an anaplerotic role by replenishing TCA cycle intermediates for the production of reducing equivalents that drive the mitochondrial respiratory chain and generate lipid and nucleotide biosynthetic precursors, it serves in the central nervous system mainly the synthesis of the neurotransmitters glutamate and GABA within the “glutamine-glutamate” cycle between astrocytes and neurons (Schousboe and Sonnewald, 2016).

Acetyl CoA, derived from glucose and ketone bodies, is the major fuel in early life to enter the TCA cycle that oxidizes acetyl CoA to carbon dioxide. In this process reduced cofactors (three molecules of NADH and one molecule of FADH_2_) are produced that are a source of electrons for the ETC. This system consists of five serially interacting protein complexes (see below) that synthesize ATP from ADP and inorganic phosphate (P_i_). Although mitochondria are the major intracellular source of vital ATP production, they cannot have ATP in stock. ATP is the universal “currency” on the cell’s energy market and is rapidly called off by numerous cellular processes. The adult human body contains as little as 60–80 g of ATP, while its daily turnover rate corresponds to 40–70 kg of *de novo* ATP synthesis. This number underscores the role of the mitochondrion as cellular power plant.

Mitochondria are interconnected at different layers with the stress response to swiftly satisfy the enhanced energy demand. Historically, GCs are well known to increase circulating glucose concentrations through actions on the liver, skeletal muscle and adipose tissue (Magomedova and Cummins, 2016). Following binding to the glucocorticoid receptor (GR), a ligand-gated transcription factor (TF), GCs induce the expression of genes in the liver that encode rate-limiting enzymes in gluconeogenesis and thus lead to increased glucose synthesis and output. In this context it is important to note that neurons are incapable of gluconeogenesis and entirely depend on peripheral sources. Additionally, GCs reduce glucose uptake in muscle and adipose tissues by inhibiting the translocation of the insulin-sensitive glucose transporter GLUT4 to the plasma membrane. In muscle, GCs also induce expression of the pyruvate dehydrogenase kinase isoform 4 (Pdk4), a mitochondrial protein, which inhibits pyruvate oxidation (Figure 3). Consequently, the inhibitory effects of GCs on peripheral glucose utilization increase passively the blood levels of glucose. Furthermore, GCs regulate multiple processes related to lipid metabolism by controlling lipolysis or adipogenesis under fasted or fed states, respectively.

Beyond the regulation of glucose availability, hormones influence mitochondrial function also directly: receptors for GCs, estrogens, androgens and for thyroxin, have been detected in the mitochondrion, or translocate from the cytoplasm into the mitochondrion upon ligand binding (Psarra et al., 2006; Du et al., 2009). In cultured neurons and in PFC tissues, treatment with low doses of GCs improved mitochondrial dynamics by protecting mitochondrial oxidation, membrane potential and Ca^2+^ holding capacity against kainic acid induced toxicity. By contrast, high or prolonged doses of GCs enhanced kainic acid induced toxicity (Du et al., 2009) suggesting that chronic stress contributes to ROS production and oxidative damage (Tang et al., 2013). In support of this hypothesis, exposure to chronic stress triggers mitochondrial damage and dysfunction in different regions of the rodent brain including the hippocampus, cortex and thalamus (Madrigal et al., 2001; Rezin et al., 2008; Gong et al., 2011). Interestingly, acute immobilization stress reduced overall mitochondrial RNA expression in rat hippocampus and led to a significant downregulation of mitochondrial genes (Figure 1) encoding NADH dehydrogenases (type 1, 3 and 6) and ATP synthase, whereas chronic stress caused a significant up-regulation of NADH dehydrogenase 6 (Hunter et al., 2016). GRs bound near to the D-loop, a region controlling replication of the mitochondrial genome, and possibly regulate gene expression via structural changes. It is also worth noting that GCs induce concomitantly the expression of genes encoded by the nuclear genome, which are necessary for mitochondrial biogenesis, and that this process requires prior replication of mtDNA. Additional studies are necessary to understand the interrelationship between GC-induced changes in mitochondrial and nuclear gene expression. In any case, GC-induced mitochondrial biogenesis will expand total cellular energy capacity.

Beyond GRs, estrogen receptors (ERα and ERβ) contribute as well to mitochondrial function by stimulating the transport of glucose into the brain with subsequent glycolysis (Rettberg et al., 2014). In the female rat brain, estrogen treatment increases the expression of PDH, aconitase (an enzyme of the TCA cycle) and ATP synthase (Figures 2, 3). Estrogen receptors translocate to the mitochondrion, increase ATP levels in primary neuronal cultures, maximize mitochondrial respiratory rate in neurons and glia, and protect against ETC inhibitors (Nilsen and Diaz Brinton, 2003). These results suggest that estrogens coordinate the response of mitochondrial enzymes and improve mitochondrial bioenergetics in the brain.

Historically, the interrelationship between energy supply and brain programming is well-supported from animal and human studies. Maternal malnutrition during famine is known to lead to a modest (<2-fold) and non-specific increase in risk for schizophrenia (Susser and Lin, 1992; St. Clair et al., 2005) consistent with an experience-dependent neurodevelopmental component in this disease (Birnbaum and Weinberger, 2017). Beyond famine, poor diet quality associates with a high prevalence of mood and anxiety disorders already in children and adolescents (Baker et al., 2017). Equally important, early obesity associates with affective disorders including anxiety and depression (Patchev et al., 2014). As of yet, the involvement of mitochondria in nutritional programming of the brain is still understudied (Thompson and Al-Hasan, 2012). Even though, brain programming via early nutrition points to a role of the mitochondrion, and possibly to a related role in ELS-induced changes in energy demand.

Taken together, stress-induced release of GCs regulates the availability of glucose fueling central mitochondrial ATP-production in order to reinstate homeostasis and to serve energy intensive adaptation programs. Thereby, mitochondria fulfill a more dynamic role in the integration of stress than commonly thought: they readily sense GCs and glucose as primary mediators of the stress response and undergo functional and morphological changes to cope with and adapt to stress.

## Mitochondrial Steroidogenesis in ELS-Dependent Brain Programming

**Figure 4:**
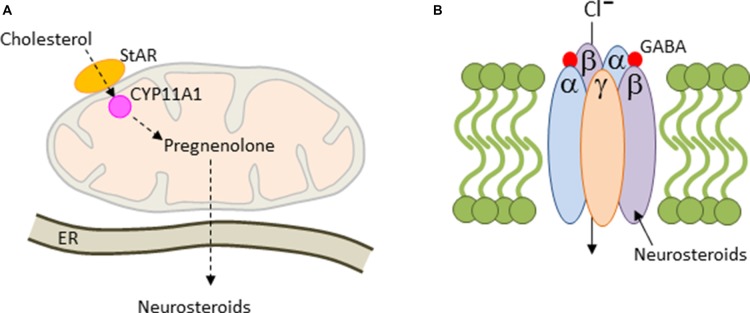
Mitochondrial steroidogenesis. **(A)** Cholesterol is translocated across the mitochondrial membrane by the steroidogenic acute regulatory protein (StAR). Thereafter, cholesterol is converted to pregnenolone by the side-chain cleavage enzyme CYP11A1. Pregnenolone is trafficked then to the endoplasmaic reticulum (ER), where it can be converted into various neurosteroids including allopregnanolone. **(B)** Most GABA_A_-receptors contain two α and β subunits each together with a single copy of the γ2 subunit. These subunits arrange around a central pore to form a chloride ion-conducting channel. Allopregnanolone is a positive allosteric regulator of GABA_A_-receptors leading to prolonged channel opening and hyperpolarization.

The *de novo* synthesis of all steroids is initiated with the translocation of cholesterol across the mitochondrial membrane by steroidogenic acute regulatory protein (StAR) and translocator protein 19 kDA (TSPO), a rate limiting step in cholesterol uptake (Figure 4A). In the mitochondrion, cholesterol is converted to pregnenolone (PREG) by the side-chain cleavage enzyme CYP11A1, a member of the P450 superfamily of proteins. CYP11A1 is hosted at the inner mitochondrial membrane and is the first and rate-limiting enzymatic step in steroid biogenesis (Payne and Hales, 2004). Thereafter, pregnenolone can be converted to various derivatives in the endoplasmaic reticulum (ER; Figure 4A). Steroid synthesis was originally thought to be confined to the adrenal cortex, the gonads and placenta, until Baulieu (1997) discovered that brain tissues are also capable of steroid synthesis, either *de novo* or via metabolism of the blood-borne precursor deoxycorticosterone, testosterone and progesterone, and referred to these steroids as neurosteroids.

Over the past 20 years, increasing evidence indicates a role of mitochondrial steroidogenesis in ELS-dependent brain programming extending from intrauterine to perinatal life and beyond, and by modulation of maternal stress responsivity.

Placental function is regulated by the concerted interaction of maternal decidual cells, trophoblastic cells and fetal endothelial cells in response to the local and systemic environment. Disruption of the maternal milieu by stress can impact vital aspects of placental structure and function, including integrity of the protective transplacental barrier, nutrient and oxygen exchange and the integrity of the fetoplacental unit (see below; Burton et al., 2016). A decade ago, Fowden et al. (2006) suggested that placental mitochondria could serve as a central hub for integrating nutritional (see previous section), endocrine (this section) and oxidative (following section) signals in intrauterine programming of child development. Along this line, he further reasoned that maternal exposure to life stress can affect lastingly placenta’s mitochondrial capacity, endanger well-being of mother and child, and mediate prenatal brain programming in the infant. Although programming of mitochondrial oxidative functions by ELS is well-recognized (see following section), programming of mitochondrial endocrine functions is still understudied, partly due to the need to conduct such studies on human placenta.

Placental endocrine functions (Costa, 2016) differ between rodents and human: in rodents, steroidogenic enzymes, such as rate-limiting CYP11A1 (hosted by the mitochondrion) are expressed in early trophoblastic giant cells, but poorly in mature placental cells. By contrast, steroidogenic enzymes, including mitochondrial CYP11A1, are well expressed in the human placenta late in gestation.

The majority of normotensive pregnancies (Aufdenblatten et al., 2009) are characterized by the absence of placental cortisol. However, cortisol is detectable in the placenta of up to 80% of pre-eclampsia associated pregnancies. This raises concern given overwhelming evidence for the role of GCs in developing programming associated with low birth weight, hypersensitive stress responses, and neurobehavioral anomalies in infants, and increased risk of metabolic, cardiovascular and psychiatric symptoms in adults (Gluckman and Hanson, 2004). Interestingly, pre-eclampsia, particularly in its more severe early onset form, leads to placental *CYP11A1* hypomethylation (Hogg et al., 2013) that correlates with increased gene expression. Thus, dysregulation of the placental epigenome in pre-eclampsia implicates genes regulating the hormonal environment during pregnancy that could offer an explanation for increased placental cortisol, at least in part.

Physiologically, the human placenta secrets estrogen and progesterone at about 10 times the amount secreted by the mid-luteal-phase corpus luteum and in much larger amounts than in other mammals. Around term, the human placenta synthesizes in the syncytiotrophoblast about 0.3 μg progesterone *per* day from maternal cholesterol through the catalytic activities of CYP11A1 and 3βHSD (hosted by the ER). Progesterone is released into the maternal compartment (local tocolytic uterine and systemic circulation) and into the fetal compartment where it is converted into fetal adrenal steroids (cortisol and DHEA). Because the placenta cannot convert progesterone into androgens, and from there, into estrogens, placental estrogen synthesis depends on androgen precursors produced by the maternal adrenal gland; this reciprocal interdependence has been conceptualized as “fetoplacental unit.”

The human placenta is also a major site of synthesis of the neurosteroid allopregnanolone (also known as 5α-pregnan-3α-ol-20-one or 3α,5α-tetrahydroprogesterone) throughout late gestation and leads to concentrations in the brain that are higher than at any time during life. The response to physical and psychological stress is strongly suppressed in both rodents and humans in late pregnancy. This so-called hyporesponsive period (van Bodegom et al., 2017) safeguards the fetus against overexposure to maternal GCs and promotes anabolic adaptations in the mother that support timely progression of pregnancy. Inhibitory opioid signaling is enhanced during this period and reduces the excitatory drive from the forebrain and hindbrain to the hypothalamic paraventricular nucleus in order to attenuate stress-induced corticotrophin-releasing hormone (CRH) expression and HPA-axis activation (Brunton et al., 2005). The inhibitory neurosteroid allopregnanolone plays an important role in this process: allopregnanolone acts as a potent positive allosteric modulator of the action of GABA at the GABA_A_ receptor (Figure 4B) by promoting channel opening, chloride ion influx and hyperpolarization of the cell membrane. Allopregnanolone has effects similar to other positive modulators at the GABA_A_ receptor such as benzodiazepines, including anxiolytic, sedative and anticonvulsant activity. In agreement with this mode of action, blocking allopregnanolone synthesis in late pregnancy reinstates HPA-axis reactivity to stress with potential repercussions on child development (Brunton et al., 2009).

Levels of allopregnanolone drop dramatically with the loss of the placenta after birth (Kelleher et al., 2013) or in the case of preterm birth, and vary considerably across postnatal development: they are low for the first postnatal week, increase again, and return to low adult levels (Grobin et al., 2004). In support of a regulatory role of these fluctuations, Zimmerberg et al. (1994, 1999) first proposed about 25 years ago a role of neurosteroids in the programming of the stress response based on the observation that administration of the neurosteroid allopregnanolone reduced signs of anxiety in neonatal rat pups previously exposed to maternal separation (MS), an ethnological relevant model of ELS (Lehmann and Feldon, 2000). Consistent with this finding, allopregnanolone significantly reduces signs of anxiety in ELS-exposed neonate rats via its effects on GABA_A_ receptors (Vivian et al., 1997). Interestingly, administration of allopregnanolone during the separation period ameliorated the behavioral and neuroendocrine effects from MS in adult rodents (Patchev et al., 1997) implicating a direct role of neurosteroids in the long-term sequelae of MS.

The postnatal brain is highly plastic due to ongoing cell migration, arborization and synaptogenesis. Beyond behavior, allopregnanolone affects at the cellular level the localization of parvalbumin-expressing GABAergic interneurons in the adult PFC (Grobin et al., 2003). A plausible mechanism for allopregnanolone is the enhancement of GABA_A_ receptor function given that ambient GABA facilitates cortical entry of tangentially migrating neurons derived from the medial ganglion eminence. ELS leads to a significant reduction in the density of parvalbumin-positive GABAergic interneurons in the medial PFC and hippocampus (Giovanoli et al., 2014; Uchida et al., 2014) as well as in the number of GABA_A_ receptors in the hippocampus and central amygdala (Fride et al., 1985; Barros et al., 2006). It is interesting to note that similar changes have been detected in schizophrenia and autism and that these disorders associate with prenatal stress exposure (Fine et al., 2014) and mitochondrial dysfunction (Srivastava et al., 2018; Sullivan et al., 2018).

Collectively, the mitochondrion controls the first and rate-limiting step in *de novo* synthesis of steroids throughout life. Mitochondrial steroidogenesis takes place in different organs (placenta, adrenal glands and neuronal cells) and serves to orchestrate the closely interwoven interactions between the developing child and the mother, both pre- and postnatally. Emerging evidence from animal studies suggests that the effects of fetal and/or maternal stress associate with altered steroidogenesis implicating, at least in part, perturbed mitochondrial function.

## Mitochondrial ROS Production in Neurodevelopment and Brain Programming

**Figure 5:**
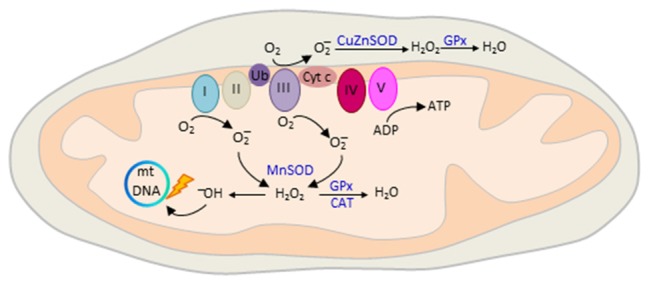
Mitochondrial reactive oxygen species (ROS) production. The mitochondrial electron transport chain (ETC) consists of five interacting protein complexes numbered I to V that build a proton gradient across the inner mitochondrial membrane. Electron leakage mainly at complex I and III reduces prematurely oxygen to superoxide anion (${\text{O}}_{2}^{-}$) during erobic respiration. ${\text{O}}_{2}^{-}$ is converted by mitochondrial superoxide dismutase MnSOD and CuZnSOD to hydrogen peroxide (H_2_O_2_) in the matrix or intermembrane space, respectively. Thereafter, H_2_O_2_ is oxidized to highly reactive hydroxyl (OH^−^) free radicals that cause mtDNA damage, reduced transcription and oxidation of ETC proteins and membrane lipids. ROS production is counteracted by enzymatic defense mechanisms including glutathione peroxidase (GPx) and catalase (CAT) that detoxify H_2_O_2_ into water. Colors from respiratory complexes match the contribution of mitochondrial gene products shown in Figure 1. Ub, ubiquinone, also known as Coenzyme Q10; Cyt c, cytochrome c.

Oxidative phosphorylation of nutrients is the major source (≈90%) of deleterious ROS production in eukaryotic cells (Hroudová and Fišar, 2013). The mitochondrial respiratory chain transports protons (hydrogen ions) across the inner mitochondrial membrane by a series of oxidation-reduction reactions with each acceptor protein owing a greater reduction potential than the foregoing (Figure 5). Though few, mitochondria genes (Figure 1) encode key structural subunits of the respiratory chain required for NADH dehydrogenase (complex I), cytochrome c reductase (complex III), cytochrome c oxidase (complex IV) and ATP synthase (complex V).

The final destination for an electron passed along this chain is an oxygen molecule (Figure 5). Under normal conditions, the oxygen is reduced to produce water; however, in about 0.1%–2% leakage of electrons mainly at complex I and III leads to the reduction of oxygen to superoxide anion (${\text{O}}_{2}^{-}$; Turrens, 2003). ${\text{O}}_{2}^{-}$ is not particularly reactive by itself, but can be converted to hydrogen peroxide (H_2_O_2_), or interact instead with nitric oxide to produce peroxynitrite. H_2_O_2_ is metabolized to highly reactive hydroxyl (OH^−^) free radicals (Figure 5) that cause broad cellular damage (Valko et al., 2007). The continuous production of ROS under physiological conditions is balanced by enzymatic and non-enzymatic cellular scavenging strategies. Among the enzymatic defense mechanisms, catalase (CAT) and glutathione peroxidase (GPx) detoxify H_2_O_2_ into water and molecular oxygen. Small molecules such as ascorbic acid and tocopherol scavenger further aid in the detoxification of free radicals.

The developing fetal brain is extremely susceptible to free radical-induced damage given its high oxygen consumption relative to the adult brain and its high basal levels of ROS production. Even more, the developing brain contains a high concentration of lipids, including a high content of polyunsaturated fatty-acids (PUFAs; see below) that are particularly prone to lipid peroxidation, while antioxidant defense mechanisms are still less developed compared to the adult (Gitto et al., 2009). Mitochondria are also particularly exposed to oxidative stress since their genome is nonchromatinized and owes fewer DNA repair mechanisms than the nucleus (Figure 5). Damage to mitochondrial proteins and membranes can initiate a positive feedback mechanism in which oxidative injury of few mitochondria triggers failure of an entire network of mitochondria (Aon et al., 2006).

Notwithstanding their deleterious effects, mitochondrial ROS also serve mitochondrial-nuclear interactions that support survival under conditions of limited nutrient availability. ROS provide a dynamic feedback mechanism for the regulation of a variety of enzymes, signaling pathways and TFs (e.g., nuclear factor κB, NFκB; nuclear erythroid 2 p45-related factor 2, Nrf2; and peroxisome-proliferator-activated receptor coactivator-1α, PGC-1α) that converge on redox-sensitive gene expression programs (Hancock et al., 2001). In light of these findings, Luo et al. (2006) suggested that programming of adult disease is mediated by oxidative stress during critical time windows of development. Oxidative stress-mediated programming could operate either directly through the modulation of gene expression or indirectly through the deleterious effects of oxidized lipids or other molecules.

Pregnancy poses a major metabolic challenge, and as such even a normal, healthy pregnancy represents a state of oxidant stress met by both the mother and her developing baby (Little and Gladen, 1999). Above all, the placenta is a major source of oxidant stress owing to its high metabolic rate and mitochondrial activity (Holland et al., 2017). Like the brain, the placenta uses mainly glucose to maintain its high pace metabolism necessary to satisfy homeostatic demands from mother and child (Vaughan and Fowden, 2016).

Many known or potential risk factors associated with intrauterine growth retardation (IUGR) or preterm birth cause oxidative stress: malnutrition, a well-known cause of poor fetal growth, can impair cellular antioxidant capacities either through an insufficient supply of amino acids needed for the synthesis of antioxidants (e.g., glutathione) or due to the absence of ingredients with an antioxidant role on their own (Herrera et al., 2009). Gestational hypertension or preeclampsia frequently cause impaired fetal growth or preterm birth and have been consistently associated with oxidative stress (Lain and Roberts, 2002). Likewise, diabetes mellitus or obesity can impair placental mitochondrial structure and function (Holland et al., 2017). Smoking is a pro-oxidative factor producing large amounts of ROS, comprising *in vivo* antioxidant capacity, and contributing to numerous poor birth outcomes, such as low birth weight, preterm birth as well as life-long health and developmental problems (Stone et al., 2014). Pregnancies complicated by severe reduction in oxygen supply to the fetus or intrauterine infection result in a state of fetoplacental oxidative imbalance. Notably, oxidative stress and proinflammatory processes are strongly interrelated (Redman and Sargent, 2003). Once activated, many cells in the immune system produce ROS, while at the same time, overproduction of ROS triggers inflammatory responses. One of the factors joining these two processes is NFκB that is upregulated in repose to both inflammations and hypoxia (Li and Karin, 1999).

Importantly, all of these conditions—hypertension, diabetes mellitus, obesity and social stress—are interconnected in gestational risk and contribute to poor fetal and postnatal outcome. Mitochondrial oxidative stress may offer a link between these risk factors, and equally, to the programming of cardiovascular, metabolic and neurobehavioral (mal) functions in the offspring.

## The Role of the Mitochondrion in ELS-Dependent Programming of Rodent Brain

In light of mitochondrion’s role in the mediation and regulation of early stress responses, we focus now on concrete examples supporting the role of mitochondrial function and ROS production in ELS-dependent programming of rodent brains. For clarity, we will discuss these studies based on a common focus rather than by their chronological order and summarize key findings in Table 1.

**Table 1:** Early-life stress (ELS)-dependent programming of mitochondrial functions in rodents.

Reference	S	Model	Tissue	Major finding
Zhu et al. (2004)	r	PRS_G7–13_ PRS_G14–20_	Hipp	Increased ROS production in CA3 region
Song et al. (2009)	r	PRS_G14–20_ PRS_G14–20_	Hipp	Enhanced ROS-dependent mitochondrial DNA damage in the CA3 region
Diehl et al. (2012)	r	MS_P1-P10_ ± ad FS	Hipp	Deficits in spatial memory, DNA breaks, unaffected antioxidants
Krolow et al. (2012)	r	JS_P21-P28_	PFC	Increased Complex IV and superoxide dismutase activities in juveniles and adults, unaltered mitochondrial mass and potential
van Zyl et al. (2016)	r	MSP_2–14_ ± ARS	PFC	Changes of inner membrane mitochondrial proteins relevant to ATP production concur with depression-like behavior
Marcelino et al. (2013)	r	ME	Hipp, PC, Cer, Stri	Programming of mitochondrial and anti-oxidants function and of mitochondrial biogenesis
Park et al. (2013)	m	ME	Brain	Programming of mitochondrial bioenergetics and biogenesis and memory functions, in the brain of the offspring
Zugno et al. (2015)	r	MS_P1-P10_ ± ad Ket	PFC, Stri, Hipp	Tissue-dependent programming of mitochondrial metabolic and oxidative functions associates with reduced NGF expression in the hippocampus
Réus et al. (2017a)	r	LPS_P9.5_ ± ad Ket	PFC, Stri, Hipp,	Immune activation potentiates ketamine- induced inflammation and oxidative stress
do Prado et al. (2016)	r	MS_P2-P20_ ± ado EE	PFC	Oxidative stress induced loss of prefrontal interneurons is unresponsive to EE
Amini-Khoei et al. (2017)	m	MS_P2-P14_ ± OXT	Hipp	Decreased mitochondrial ATP and increased ROS production is rescued by oxytocin
Feng et al. (2012)	r	PRS_G14–20_ ± DHA MS_P1-P10_	Hipp	Maternal DHA administration prevents behavioral impairments, oxidative damage and mitochondrial dysfunction in offspring
Ferrari et al. (2016)	r	± ω-3FA MS_P1-P10_	Hipp	Early-life stress and DHA deficits increase susceptibility to oxidative stress
Réus et al. (2018)	r	CMS ± AntiOx	PFC, Stri, Hipp, Amy	Antioxidant treatments prevent depressive-like behavior and oxidative changes from early or adult stress

*Abbreviations: ad, adult; ado, adolescent; Amy, amygdala; AntiOx. ω-3 PUFA, folic acid or n-acetylcysteine; ARS, adult restraint stress; Cer, cerebellum; CMS, chronic mild stress from postnatal day 40–100; d, day; DHA, supplementation (+) or deficits (−) in docosahexaenoic acid; EE, environmental enrichment, FS, foot shock; Hipp, hippocampus; G, gestational day; JI, juvenile isolation; Ket, ketamine, LPS, lipopolysaccharides; ME, maternal exercise; MS, maternal separation, NGF, nerve growth factor; ω-3 FA, omega-3 polyunsaturated fatty acids; P, postnatal day; PFC, prefrontal cortex; PRS, prenatal restraint stress; S, species. Stri, striatum*.

### Different Qualities of Stress

The involvement of oxidants in the stress response has been hypothesized some 20 years ago (McIntosh and Sapolsky, 1996; Liu and Mori, 1999); still, it was not before 2004 when Zhu et al. (2004) explored their role in ELS using a model of restraint stress treatment of rat dams (Table 1). At 1-month age, hippocampi from prenatally stressed offspring showed a loss of neuronal cells and increased ROS production in the hippocampal CA3 region. Well-fitting, a follow-up study (Song et al., 2009) reported an increased 8-Hydroxy-2′deoxyguanosine (8-OH-dG) content, a reaction product from ROS, in the mtDNA from rat hippocampi.

To study the role of ELS in predisposing to posttraumatic stress disorder (PTSD), Diehl et al. (2012) exposed neonate rats to MS and re-exposed them in early adulthood to foot shock (FS), a stressor commonly used to elicit PTSD-like behavior. Rats with a history of MS or shock showed long-lasting deficits in spatial memory and more DNA strand breaks in the hippocampus. DNA damage was more pronounced under combined stress treatment and is a likely proxy to oxidative damage. In a related study, Krolow et al. (2012) investigated the effects of isolation stress in prepubertal rats on oxidative stress and energy metabolism in prefrontal cortices of juvenile and adult male rats. Prepubertal stress increased mitochondrial complex IV and superoxide dismutase activities in juvenile and adult cortices. However, mitochondrial mass and potential, free radical production and antioxidant activities were unaltered. Additional studies are warranted to understand the relevance of increased complex IV activity in the presence of unaltered mitochondrial numbers and overall activity.

van Zyl et al. (2016) further asked whether superimposition of adult stress could exacerbate the effects of early stress in rats (Table 1). MS induced depression-like behavior with superimposition of adult restraint stress revealing no additional effects. Subsequent proteomic analysis of prefrontal cortices evidenced a decrease in mitochondrial energy-related proteins in early, adult and combinatorial stress-treated rats with combinatorial stress leading to an additional decline in mitochondrial protein synthesis concomitant to an increased expression of proteins relevant to protein degradation.

Collectively, different qualities of stress, especially ELS, can lead to lasting alterations in mitochondrial bioenergetic functions and antioxidant mechanisms across brain regions relevant to cognition, emotion and behavior.

### Physical Exercise

Physical exercise, as opposed to maternal stress, is known to benefit both mother and child. Marcelino et al. (2013) studied in rat the effect of maternal swimming on five brain regions (Table 1) from newborn offspring. Maternal exercise (ME) led to reduced mitochondrial superoxide (O_2_) and increased reactive species (mainly nitric oxide, RNO) with some variation across tissues. These changes concurred with an improved antioxidant status in both enzymatic and non-enzymatic parameters. Interestingly, mitochondrial mass and membrane potential were increased in the neonate parietal cortex and cerebellum indicative of mitochondriogenesis.

In support of these findings, Park et al. (2013) observed that ME (Table 1) during pregnancy affects mitochondrial enzyme activity and biogenesis in mice neonate brains dependent on the duration of the exercise: ME enhanced the expression of the inner mitochondrial membrane complex Va and of the F1-ATPase catalytic core, while molecules from the outer mitochondrial membrane were unchanged. Furthermore, the activity of PDH, of enzymes regulating ATP production and of inner membrane redox carriers was likewise increased. Concurrently, increased lipid peroxidation indicated that changes in mitochondrial enzyme expression and activity converged on ROS production. Interestingly, mitochondrial biogenesis was also increased in neonate mice brain as suggested by increased levels of PGC-1α, Nrf-1, mitochondrial Tfam (mitochondrial TF A, a key activator of mitochondrial transcription and a participant in mitochondrial genome replication) and of mtDNA content. Regarding behavior, ME improved short-term memory formation in adolescent offspring concurrent to increased hippocampal brain derived neurotrophic factor (BDNF) expression.

Taken together, ME can lastingly enhance mitochondrial bioenergetics and biogenesis in offspring brain with positive effects on memory function.

### Early Stress and Schizophrenia

ELS is a well-recognized risk factor for schizophrenia that sensitizes adult rats to acute treatment with ketamine, a noncompetitive NMDA receptor antagonist, as evidenced by increased hyperlocomotion and social contact latency (Zugno et al., 2013). Zugno and coworkers analyzed in rat PFC, hippocampus and striatum: (i) creatine kinase (CK, a mitochondrial enzyme catalyzing the formation of phospho-creatine (PCr) that serves as an energy reservoir for the rapid regeneration of ATP in tissues with high energy demand); (ii) enzyme activities of the TCA cycle; (iii) mitochondrial respiratory chain complexes; (iv) lipid peroxidation and protein carbonylation; and (v) neurotrophic factor levels (nerve growth factor (NGF) and BDNF). This comprehensive analysis was carried out on untreated adult rats or adult rats exposed to MS, ketamine, or both conditions. All treatments reduced hippocampal CK activity and increased striatal TCA activity. Furthermore, combined treatment increased the activity of mitochondrial chain complexes to varying degree across all tissues and correlated with increased prefrontal protein carboxylation. In the hippocampus, MS alone or in combination with ketamine reduced NGF levels, while BDNF was unaffected.

Immune activation (IA) during early life is a predisposing factor for the development of schizophrenia and can be mimicked in rats by treatment with lipopolysaccharides (LPS, molecules found on the outer membrane of Gram-negative bacteria). To investigate the role of IA in ketamine-induced schizophrenia-like behavior and oxidative-stress induced damage and neuroinflammation, Réus et al. (2017b) injected neonatal rats with LPS and administered the adult offspring low or high doses of ketamine known to lack or to set-off schizophrenic-like behavior. Levels of cytokines and oxidative stress markers were determined in the PFC, hippocampus and striatum. On its own, IA failed to induce hyperlocomotion, brain oxidative stress or inflammation. By contrast, a high dose of ketamine evoked hyperlocomotion irrespective of the immune history. Interestingly though, previous IA exacerbated the effect of ketamine on oxidative stress and inflammation in brain regions relevant to schizophrenia.

Taken together, these studies suggest that ELS as early risk factor for schizophrenia can program in a brain region-specific manner enhanced mitochondrial metabolic and oxidative functions that enhance ketamine-dependent effects. Thus, a first insult may program cellular endophenotypes more sensitive to subsequent oxidative insult and potentiate its effects.

### Environmental and Pharmacological Interventions

The effect from MS on the PFC typically manifests in adolescence due to its late and protracted development and associates with decreases in parvalbumin-containing GABA-ergic inhibitory interneurons (Holland et al., 2014). ROS derived from mitochondria or from NADPH oxidases (NOX, a group of cytoplasmatic proteins that transfer electrons across biological membranes to produce superoxide) have been implicated in this process (Schiavone et al., 2016). do Prado et al. (2016) exposed MS rats to environmental enrichment (EE; Vivinetto et al., 2013) that improved cognitive dysfunctions in MS male and female rats and prevented elevated circulating pro-inflammatory cytokines in males, but not in females. By contrast, EE did not protect against parvalbumin loss or adolescent measures of oxidative stress, whereby adolescents with less PFC parvalbumin and higher oxidative damage showed more deficits in short- and long-term memory tasks.

More recently, Amini-Khoei et al. (2017) asked whether MS could program mitochondrial function in the hippocampus, a key target and regulator of stress, and whether this event could be ameliorated by pharmacological treatment with the neuropeptide oxytocin. Male mice, naïve or maternally separated (Table 1), were each subdivided in four experimental groups that were treated once with saline, oxytocin, an oxytocin antagonist (atosiban), or oxytocin plus atosiban near the time to behavioral phenotyping and brain collection. Saline-treated MS-mice displayed depression-like behavior known to be induced in response to ELS. Notably, application of oxytocin, a neuropeptide regulating social, reproductive and stress behaviors (Shamay-Tsoory and Young, 2016), attenuated the behavioral effects from programming. Furthermore, in support of oxytocin’s specific effect, administration of atosiban increased depressive-like behavior in mice without a history of MS, whereas co-treatment with atosiban and oxytocin showed no significant effect.

Moving beyond behavior, Amini-Khoei et al. (2017) analyzed mitochondrial ATP and ROS production in hippocampi from naïve or MS-treated mice for each experimental subcondition. Interestingly, MS led to reduced mitochondrial ATP production relative to naïve mice, which was normalized by application of oxytocin. Vice versa, MS increased mitochondrial ROS production relative to naive controls, which was strongly reduced by application of oxytocin. In naïve mice, oxytocin showed no significant effect on both hippocampal ATP and ROS production, whereas atosiban slightly reduced ATP production and strongly increased ROS production. Well-fitting this result, atosiban strongly enhanced the effects from MS on both mitochondrial ATP and ROS production.

At the molecular scale, increases in mitochondrial ROS production associated with increased expression of different immune-modulatory genes in the hippocampus that are potential downstream effectors of MS-dependent programming of brain function. Changes in hippocampal gene expression were attenuated, or even normalized, by oxytocin treatment of MS mice, which showed no effect in naïve mice.

In conclusion, these studies show that ELS-dependent programming of cognitive dysfunction and depression-like behavior extends to programming of key functions of the mitochondrion; namely ATP and ROS production. These alterations may contribute to the loss of prefrontal inhibitory interneurons and increased expression of immune-modulatory genes in the hippocampus; both of these events are relevant to ELS-induced behavioral phenotypes. Finally, part of the ELS-dependent effects on mitochondrial dysfunction were attenuated, or even normalized, by EE or treatment with the neuropeptide oxytocin.

### Polyunsaturated Fatty Acids

The developing brain contains a high percent of long-chain polyunsaturated fatty acids (PUFAs) such as arachidonic acid (AA) and docosahexaenoic acid (DHA). Both are derived from essential fatty acids that must be absorbed from diet and lie beneath concerns for brain development in the smallest preterm infants, who do not benefit from placental transfer of PUFAs during the third trimester of gestation (McNamara and Carlson, 2006). Dietary deficiency in PUFAs alters the phospholipid content of neuronal, glial and mitochondrial membranes and thus affects the structure and function of many proteins anchored on or embedded in the membrane, such as receptors, enzymes and transporters (Bourre, 2006). Rodents exposed to lasting deficits in ω-3 PUFA manifest reduced attention and learning concomitant to the development of depression-like behavior, anxiety and aggression (Catalan et al., 2002; Fedorova and Salem, 2006). Among PUFAs, DHA-phospholipid is particularly enriched in mitochondrial membranes and promotes mitochondrial biogenesis through the modulation of genes associated with energy metabolism and ATP production (Brenna and Diau, 2007; Nakamura et al., 2014). Conversely, ROS target preferentially PUFAs in mitochondrial membranes causing membrane depolarization and impaired mitochondrial function.

Feng et al. (2012) tested whether a diet enriched in DHA could prevent prenatal stress-induced behavioral and molecular alterations in the hippocampus of rat offspring. Dams were exposed to prenatal restraint stress (PRS; Table 1) and DHA was applied at low or high doses for 2 weeks. As expected, restraint stress caused reduced learning and memory formation concurrent with decreased BDNF expression. Furthermore, restraint stress enhanced protein oxidation and nitric oxide synthase expression, impaired mitochondrial respiratory chain complex activity, and associated with alterations in fusion/fission and autophagy protein expression in offspring hippocampi. Interestingly, administration of DHA prevented prenatal stress-induced cognitive impairments and changes in mitochondrial dynamics in both male and female offspring.

In an complementary approach, Ferreira et al. (2015) studied the effects from the interaction between ELS and dietary deprivation of ω-3 PUFA on mitochondrial function and oxidative stress in rat hippocampus. Non-handled, handled or MS rat pups were fed postnatally until periadolescence (Table 1) diets either adequate or deficient in ω-3 PUFA. Chronic deficits in ω-3 PUFA increased antioxidant enzyme activities, free radical production and thiol content primarily in the hippocampus of animals exposed to neonatal manipulations. Furthermore, reduced mitochondrial potential was detected only in animals with a history of neonatal stress. Given the absence of detectable hippocampal oxidative damage in the presence of increased antioxidant enzyme levels, the authors proposed that these increases may represent an attempt to compensate for increased mitochondrial ROS production.

By extending on potential treatments, Réus et al. (2018) recently reported the effects of ω-3 PUFA, folic acid and n-acetylcysteine (NAC) on rats exposed to early, adult, or combinatorial stress (Table 1). Folic acid is an essential vitamin administered during pregnancy to prevent neural tube defects, while NAC, a treatment for schizophrenic and mood disorders, is hypothesized to exert beneficial effects through its modulation of glutamate and dopamine neurotransmission and its antioxidant properties (Berk et al., 2013). Behavioral testing and redox measurements on brain tissues (Table 1) at day 60 (MS group) or at day 100 (chronic mild stress, CMS alone or combinatorial stress) revealed that depressive-like behavior induced by adult CMS was prevented by folic acid and NAC. Interestingly, depressive-like behavior due to ELS was additionally prevented by ω-3 PUFA application strengthening its critical role during postnatal development. Furthermore, all preventive treatments exerted antioxidants effects in the brain of rats subjected to ELS or chronic adult stress by decreasing levels of lipid peroxidation, protein carbonylation and antioxidant enzymatic activities.

Collectively, prenatal stress-dependent programing of offspring behavior and mitochondrial function can be normalized by a maternal DHA-rich diet. Relatedly, neonatal interventions can program altered susceptibility to oxidative stress (Ferreira et al., 2015) or overt oxidative dysregulation (Feng et al., 2012) in a manner dependent on the severity of the respective intervention. In any case, antioxidant treatments prevent behavioral and oxidative changes that arise from different qualities of stress exposure, whereby ω-3 PUFA is particularly effective against ELS reflecting its important role in early brain development.

## The Role of the Mitochondrion in ELS-Dependent Programming in Human

### Placental and Cord Blood Mitochondria

Fetal brain development is deeply interwoven with maternal psychosocial stress in pregnancy that constitutes an important, albeit mechanistically poorly understood factor for infant development (Zijlmans et al., 2015). Lambertini et al. (2015) investigated the expression of protein-coding mitochondrial genes in the placenta from mothers exposed to psychosocial stress in pregnancy and the relation to infant temperament. Three genes (*MT-ND2*, *MT-ND6* and *MT-CO2*) belonging to OXPHOS (Figures 1, 5) correlated to both maternal stress and infant temperament indices with *MT-ND2* showing the strongest associations: maternal psychosocial stress, known to negatively correlate with infant temperament, led to an increase in *MT-ND2* expression, which in turn correlated negatively with infant temperament.

In a recent study, Brunst et al. (2017) investigated the role of mitochondria in the mediation of programming by referring to previous evidence that placental mtDNA copy number can respond to environmental exposures that induce oxidative stress (Shaughnessy et al., 2014). To evaluate maternal stress during pregnancy, the researchers implemented an integrated analysis that assessed maternal lifetime stressors, current negative life events and measures of current psychological functioning, including depressive symptoms and PTSD. This approach informed on the cumulative stress load and allowed insight into which component(s) contribute most significantly to reduced placental mtDNA content. Although all measures of stress were associated with decreased placental mtDNA content, higher lifetime stress and depressive symptoms were better predictors of reduced mtDNA content than the measure of prenatal negative life events.

In a follow-up study, Brunst et al. (2018) investigated the interrelationship between gestational exposure to ambient fine particulate matter (PM), a physical stressor and maternal social stress during pregnancy on placental and cord blood mtDNA copy numbers. The umbilical cord joins the fetus to the placenta and is physiologically and genetically part of the fetus. Peripheral blood mononuclear cells (PBMCs) collected from the umbilical cord are thus of fetal origin.

Recent studies have shown that increased exposure to PM during the 3rd trimester of pregnancy or in late pregnancy (35–40 weeks gestation) associated with reduced mtDNA content in placenta (Janssen et al., 2012) or cord blood, mainly among boys (Rosa et al., 2017). Consistent with this data, Brunst and coworkers found that: (i) prenatal PM exposure decreased mtDNA content in placenta and cord blood; (ii) maternal stress decreased placenta mtDNA content; and (iii) prenatal PM exposure and placenta mtDNA content associated positively in boys whose mothers had experienced high lifetime stress.

Considering these studies together, changes in placental mitochondrial gene expression and mtDNA copy numbers in response to maternal stress strengthen the critical role of the mitochondrion in programming (neuro-) development during pregnancy and beyond. Hereby, a life-course perspective on maternal stress appears more informative than a focus on stress experienced rather close to pregnancy. In any case, maternal stress seems to regulate mtDNA content preferentially in the placenta, possibly due to an effect on metabolism, while physical stressors appear to act on both placenta and cord blood.

### Buccal and PBMC Mitochondria

In a large-scale study, Cai et al. (2015) reanalyzed a cohort of Chinese women that had been originally recruited for the identification of major depressive disorder (MDD) risk genes (CONVERGE Consortium, 2015). This cohort comprised 5,864 women with recurrent MDD and 5,783 matched controls whose whole genome low coverage sequencing data from buccal cells and aggregate measures of lifetime adversities, including childhood maltreatment, had been obtained. In the current study, the researchers focused on mtDNA content and telomere length (dynamic nucleoprotein-DNA structures that maintain linear chromosome ends, i.e., telomeres and shorten progressively with each cycle of DNA replication). Interestingly, MDD cases showed significant increases in mtDNA content concomitant with reduced telomere length. The amount of mtDNA was significantly correlated with both the total number of stressful life events and childhood sexual abuse, while telomere length was significantly shorter under either condition. Moreover, both molecular markers associated more strongly with increasingly severe childhood sexual abuse. These associations were unrelated to antidepressant treatment or changes in the samples’ cellular composition. Furthermore, findings on MDD and increased mtDNA content could be replicated in a European case-control study comprising both sexes. Importantly though, changes in mtDNA content and telomere length in individuals with or without adverse early life experiences were contingent on the depressed state indicating that early life adversity (ELA) confers an increased susceptibility to later stress-related disorders.

Relatedly, Tyrka et al. (2016) showed that childhood adversity and lifetime psychopathology each associated with higher mtDNA copy numbers in PBMCs. Significantly higher mtDNA copy numbers were detected in individuals, who had experienced parental loss, childhood maltreatment, MDD, depressive or anxiety disorders, or substance abuse. Changes in mtDNA content correlated significantly with shorter telomere lengths across each diagnostic category consistent with above findings (Cai et al., 2015) and numerous studies that suggested that oxidative stress caused by inflammation, intrinsic cell factors, environmental exposures and stress concurs with accelerated telomere shortening and dysfunction (Barnes et al., 2018).

The role of ELS for oxidative damage and antioxidant regulation has been further collaborated by do Prado et al. (2016) in a study on adolescents with a history of maltreatment but without manifest psychiatric disease: measurement in plasma samples showed that childhood maltreatment associated with higher levels of oxidative markers concomitant with reduced antioxidant activities.

A recent study by Boeck et al. (2016), has provided further insight into the role of the mitochondrion in these processes. Individuals with a history of child maltreatment are not only at an increased risk for psychiatric disorders later in life, but also for an increased prevalence of inflammation-associated disorders such as immune dysregulation, cardiovascular diseases and cancer (Meyer et al., 2018). Therefore, the researchers analyzed pro-inflammatory markers, serum oxidative stress markers and PBMC mitochondrial function in adult women with a history of early maltreatment. Early adversity concurred with higher cellular ROS production, decreased NOS activity and reduced levels of circulating antioxidants. At the same time, oxidative stress levels corresponded to the severity of prior maltreatment exposure and associated with higher oxygen consumption and ATP production indicating an enhanced basal energy demand of peripheral immune cells. The increased energy demand of PBMCs associated with higher release of pro-inflammatory cytokines and inflammasome activation dependent on severity of maltreatment exposure.

By extending on this work, Boeck et al. (2018) investigated the interaction between serum cortisol and plasma oxytocin levels, and PBMC oxygen consumption, in postpartum mothers with a history of childhood maltreatment. Higher oxytocin levels associated with decreased immunocellular oxygen consumption related to basal mitochondrial respiration and ATP turnover, whereas higher cortisol levels showed opposite associations. Both kinds of associations were detected only among women with exposure to severe childhood maltreatment. The increase in cellular oxygen consumption indicates that peripheral immune cells have to satisfy a higher energy demand with increasing maltreatment burden and that the positive associations with higher cortisol levels may sustain the energy supply necessary for immune cell function under high stress conditions. Recall that GRs can translocate into the mitochondrion and modulate mitochondrial activity dependent on the dosage and duration of GC exposure.

Given that exposure to environmental toxicants is known to alter mtDNA methylation in the placenta (Janssen et al., 2015), Lapp et al. (2018) most recently investigated whether exposure to ELA could induce epigenetic marking of the mitochondrial genome. Direct binding of the GR to the mitochondrial genome (Hunter et al., 2016) seems to have the greatest effects on transcription of the NADH dehydrogenase 6 gene (*MT-ND6*; Figure 1). Interestingly, adults with a high burden of adverse childhood experiences revealed higher methylation across six CpG sites near *MT-ND 6* that shows the greatest transcriptional response to stress and corticosteroid treatment in rats (Du et al., 2009; Hunter et al., 2016) and is differentially methylated in those individuals with the highest exposure to childhood adversity.

In sum, convergent lines of evidence suggest that oxidative stress may mediate the effects from ELS and psychopathology on higher mtDNA numbers and shortened telomeres in peripheral cells. The finding that ELS can program mitochondrial function contingent on MDD (Cai et al., 2015) or on its own (Tyrka et al., 2016) may relate to the source of the analyte: buccal cells contained in saliva are distinct from immune cells from whole blood and latter appear particularly sensitive to the effects of oxidative stress and the programming of mitochondrial function. In support of this view, childhood maltreatment associates with sustained increases in mitochondrial oxygen consumption, ROS production, and the release of pro-inflammatory cytokines from the peripheral immune system that may fuel inflammation and feedback then on mitochondrial function (López-Armada et al., 2013). Hence, altered mitochondrial oxidative function of immune cells may bridge early maltreatment and inflammation and may be further modulated by serum oxytocin and GC levels. Interestingly, ELS can also lead to lasting epigenetic changes at the mitochondrial genome that may contribute to altered mitochondrial function and associated stress pathologies.

## Future Directions

Convergent evidence from basic processes underpinning the stress response and from experimental animal and human studies suggests that the mitochondrion plays a pivotal role in ELS-dependent brain programming and is at the same time a critical substrate for ELS. Enduring changes in mitochondrial function can bridge ELS and neurodevelopment and thus predispose to altered neurobehavioral phenotypes, and ultimately, risk for later psychiatric disorders. Thus, the mitochondrion emerges as critical intersection point connecting ELS, brain programming, mental well-being and disease.

Prenatal and postnatal stress of mother and the developing child can associate with changes in oxidative processes that prime later psychopathology or exacerbate the response to additional stressors. On a cautionary note, not all of the referred studies provide conclusive evidence for a role of the mitochondrion in these processes, but rely in part on surrogate measurements such as cytokine levels or the expression of immune-modulatory genes. Furthermore, human studies are limited to peripheral tissues that can only serve as a proxy to mitochondrial function in the living brain. By contrast, animal models provide convenient access to brain tissues across broadly varying developmental stages and allow implementation of standardized operating protocols for ELS procedures reducing thus variability among samples relative to humans. In light of these advantages, future studies need to investigate more directly ELS-dependent programming of mitochondrial functions that varies by brain region and in response to the quality of the early life stressor (Tables 1, 2).

**Table 2:** ELS-dependent programming of mitochondrial functions in human.

Reference	Condition	Tissue	Major finding
Lambertini et al. (2015)	Maternal social stress	Placenta	Mitochondrial gene expression associates with maternal stress and infant temperament
Brunst et al. (2017)	Maternal social stress	Placenta	Mitochondrial copy number is reduced by maternal stress, particularly by life time
Brunst et al. (2018)	Maternal environmental + social stress	Placenta cord blood	Mitochondrial copy number is more strongly reduced in both tissues in boys, but not girls, whose mothers experienced both stressors
Cai et al. (2015)	ELA ± MDD	Saliva or PBMC	Increased mitochondrial DNA and reduced telomeres in individuals with or without ELA are contingent on depressed state
Tyrka et al. (2016)	ELA, MDD, anx, sub.ab	PBMC	Higher mtDNA copy numbers and shortened telomeres
do Prado et al. (2016)	ELA	PBMC	Increased oxidative damage and altered antioxidant regulation
Boeck et al. (2016)	ELA	PBMC	Increased immune cell mitochondrial activity and ROS associates with inflammation
Boeck et al. (2018)	ELA + PP	PBMC	Mitochondrial activity associates positively with cortisol, but negatively with oxytocin
Lapp et al. (2018)	ELA	PBMC	Higher methylation across six CpG sites near *MT-ND6*

*Abbreviations: anx, anxiety disorder; MDD, major depressive disorder; PBMC, peripheral mononuclear blood cells, PP, postpartum; ELA, early life adversity (parental loss, physical or emotional maltreatment/neglect); sub.ab, substance abuse*.

In this regard, a recent study (Hollis et al., 2015) on trait anxiety in rats has applied pharmacological agents to support a causal role of the mitochondrion in social subordination. High-anxious animals, prone to subordination during a social encounter with a low-anxious rat, showed reduced mitochondrial complex I and II proteins and respiratory capacity concomitant with decreased ATP and increased ROS production in the nucleus accumbens. Intra-accumbal microinfusion of specific mitochondrial complex I or II inhibitors reduced social rank in less-anxious rats, thus mimicking the high-anxiety phenotype, whereas microinfusion of nicotinamide, an amide form of vitamin B3 known to stimulate brain energy metabolism, prevented the manifestation of subordinate behavior in high-anxious animals. Importantly, these pharmacological agents did not regulate other aspects of social behavior and were specific to the nucleus accumbens, as infusion in other brain regions had no effect on social dominance.

Alternatively, recent studies have shown that mitochondria can transfer between stem cells via various contact modes, comprising cell fusion, junction and tunneling nanotube formation (Wang et al., 2018). In the same line, Robicsek et al. (2013) found that *in vitro* isolated active normal mitochondria (IAN-MIT) can enter various cell types without any manipulation and stay active. Interestingly, intra-PFC injection of IAN-MIT prevented mitochondrial membrane reductions and attentional deficits in adolescent rats with a history of prenatal IA, a valued model for schizophrenia. Given these methodological advancements, future studies are warranted to inform more directly on cause-effect-relationships between ELS-dependent programming of brain function and mitochondrial dysfunction.

Our focus on selected mitochondrial functions comprising bioenergetics, steroidogenesis and ROS formation is likely to underestimate the complexity of closely interwoven interactions with the ER (Ca^2+^ homeostasis), cellular signaling, nuclear transcription and epigenetics. Epigenetic regulation of gene expression is critical in early life programming (Murgatroyd et al., 2010; Murgatroyd and Spengler, 2011b) and mitochondria (Gut and Verdin, 2013; Shaughnessy et al., 2014) are crucial for providing substrates like acetyl CoA, S-adenosylmethionine (SAM), α-ketoglutarate, ATP and NAD^+^ for epigenetic processes. Deficits in epigenetic substrates (Guéant et al., 2013) can modify different aspects of epigenetic programming, and vice versa, supplementation of epigenetic substrates (Weaver et al., 2005) can modify, or even overwrite the effects of early life programming. Although our understanding of ELS-dependent programming of mitochondrial function in the brain is still in its infancy, emerging evidence from mitochondrial bioenergetics and steroidogenesis warrants future research on mitochondrion’s capacity to provide critical substrates for epigenetic regulation in response to ELS. At the same time, a most recent study (Lapp et al., 2018) has provided compelling evidence that mtDNA in itself can serve as a substrate for epigenetic programming in response to ELA. Despite this remarkable progress, the role of the mitochondrion as intersection point in ELS is still understudied and future experiments need to progress from the current research focus to mitochondrial functions unexplored so far.

With these limitations in mind, we note that several studies aimed to improve the sequelae of altered mitochondrial function following from ELS by dietary or environmental interventions. There is increasing evidence that an imbalance in pro- and antioxidant capacity in the developing brain takes place very early in progress toward (mal-) programming and irreversible brain damage. This raises the elating perspective that targeting this imbalance may be a rewarding approach to ameliorate the long-term effects from ELS. Needless to say, any potential regiment to protect the fetus by pretreatment of the mother or of the newborn child, must be in itself benign, and impose no additional risk burden. In this regard, a number of antioxidants used in perinatal medicine for the treatment of severe chronic fetal hypoxemia are not free of potentially damaging side effects (Miller et al., 2012; Ben-Shachar and Ene, 2018). Still, other options warrant closer consideration.

In animal models, supplementation with PUFA has been reported to ameliorate the effects of ELS-dependent programming of behavioral and mitochondrial functions (Feng et al., 2012; Ferrari et al., 2016; Réus et al., 2018). As by now, randomized clinical trials that compare different maternal or infant intakes of ω-3 PUFAs or combinations of ω-3 and ω-6 fatty acids have not led to firm conclusions about the optimal PUFA status of pregnant women or infants (Demmelmair and Koletzko, 2015). In this regard, studies including mothers at risk for gestational complications and/or with a high life time stress burden may be worth considering.

Similarly, the use of NAC to ameliorate the long-term effects from ELS (Réus et al., 2018) is presently still unexplored in human. NAC is thought to target glutamatergic and dopaminergic transmission, neurotrophins, the antioxidant glutathione, inflammatory pathways and mitochondrial function (Berk et al., 2013) and thus to protect white matter integrity in early psychosis patients (Klauser et al., 2018). Early research into NAC supplementation in pregnancy suggests it may have potential as neuro- and cardioprotective agents in fetal growth restriction with no data available yet on maternal stress exposures (Groom and David, 2018).

By contrast, exercise training is well known to improve human health, including physical state, cognition, attention, emotion and resilience to stress. Exercise has been proposed for treatment for mitochondrial disorders (Hassani et al., 2010) and chronic disorders with bioenergetic failure comprising metabolic, cardiovascular and neurodegenerative disorders among others. Exercise increases mitochondrial protein mass, TCA cycle activity, ATP production and mitochondrial biogenesis (via PPAR/PGC1-α activation; Ben-Shachar and Ene, 2018). As single or add-on treatment, exercise training has moderate antidepressant and anxiolytic benefits in affective or schizophrenic disorders and in mild cognitive impairment (Zschucke et al., 2013; Hearing et al., 2016). Consistent with these findings, ME in rodents induced programming of mitochondrial anti-oxidant functions, bioenergetics and biogenesis in brain regions relevant to ELS and psychiatric disorders (Marcelino et al., 2013; Park et al., 2013). Interestingly, patients with MDD showed dependent on the severity of ELA (Cai et al., 2015) increased mitochondrial copy number that in general is considered to be beneficial. Likewise, childhood adversity and lifetime psychopathology (Tyrka et al., 2016) each associated with higher mtDNA copy numbers in PBMCs. A possible explanation for this “paradox” is that patients with MDD (de Kloet et al., 2005), but also with a history of early life trauma (Nemeroff, 2016) frequently exhibit hypercortisolemia that may induce the expression of nuclear genes regulating mitochondrial biogenesis. Whether this reflects a pathophysiological or compensatory response requires further studies. We also note that maternal social stress (Brunst et al., 2017, 2018) reduces mitochondrial copy number in the placenta and cord blood. Beyond differences in tissue type and developmental age, it is tempting to speculate that severity of hypercortisolemia may act as a critical determinant in mitochondrial biogenesis. Along this line, low doses of GCs (Du et al., 2009) improved mitochondrial function in PFC cultures, whereas high doses caused mitochondrial damage. Such dynamic regulation of mitochondrial function could refer as well to mitochondrial biogenesis and future studies are necessary to test this hypothesis. If yes, moderate rather than excessive, exercise training is envisioned to benefit mothers with high life time stress load, their up growing children and children exposed to ELA.

An intriguing possibility is the therapeutic use of oxytocin, given its beneficial effect on mitochondrial dysfunction in ELS-treated rodents (Amini-Khoei et al., 2017), and its negative associations with immunocellular oxygen consumption in postpartum mothers with a history of childhood maltreatment (Boeck et al., 2018). Oxytocin has a seminal role in mediating social affiliation, attachment, social support, maternal behavior and trust, as well as protection against stress and anxiety. Consistent with these findings, a history of child maltreatment in medically healthy women associates with decreased cerebrospinal fluid concentrations of oxytocin, particularly in cases of emotional abuse, indicating that alterations in central oxytocin function may underlie the adverse outcomes of childhood adversity (Heim et al., 2009). The oxytocinergic system has been suggested as potential pharmacological target in psychiatric disorders to ameliorate symptoms of social dysfunction in autism, social anxiety, borderline personality disorder, and schizophrenia (Kirsch, 2015) and may also serve as target in patients with a history of ELA.

While translating prevention of ELS-dependent programming of mitochondrial function in rodents into clinical trials is a daunting task, we realize that a most recent study (van der Kooij et al., 2018) found that well-established clinically used drugs such as diazepam can regulate mitochondrial functions in a site-specific manner. Benzodiazepines, the prototypic wide-spectrum anxiolytic drugs, are widely used for the treatment of social disturbances in clinical anxiety and promote social competitiveness in several species. In rats, van der Kooij et al. (2018) identified the ventral tegmental area as a major site of diazepam action in promoting social dominance with the nucleus accumbens as a critical effector region. Microinfusion of diazepam in the ventral tegmental area enhanced mitochondrial respiration in the nucleus accumbens reinforcing its role (Hollis et al., 2015) in social rank attainment in rats. Likewise, systemic diazepam treatment facilitated social dominance in high-anxious rats and highlighted that site-specific mitochondrial dysfunction is causally implicated in the beneficial effects of diazepam.

All in all, the role of the mitochondrion as a critical intersection point connecting ELS, brain programming, and mental well-being may not only provide new insight into the pathophysiology of mental illness, but also offer new therapeutic perspectives to improve the lives of patients and their families.

## Author Contributions

AH and DS wrote jointly the manuscript and carried out the artwork. DS performed the literature research.

## Conflict of Interest Statement

The authors declare that the research was conducted in the absence of any commercial or financial relationships that could be construed as a potential conflict of interest.
